# Effects of parity order on performance, metabolic, and hormonal parameters of grazing beef cows during pre-calving and lactation periods

**DOI:** 10.1186/s12917-021-03019-0

**Published:** 2021-09-25

**Authors:** Matheus Fellipe de Lana Ferreira, Luciana Navajas Rennó, Isabela Iria Rodrigues, Edenio Detmann, Mário Fonseca Paulino, Sebastião de Campos Valadares Filho, Hudson Caio Martins, Samira Silveira Moreira, Daniela Silveria de Lana

**Affiliations:** Department of Animal Science, Universidade Federal de Viçosa, Peter Henry Rolfs Avenue, Viçosa, Minas Gerais 36570–900 Brazil

**Keywords:** Blood total protein, *Bos indicus*, IGF-1, Gestation, Physiology

## Abstract

**Background:**

Metabolic profile evaluation is a tool widely used in ruminant nutrition as metabolic cues that relate nutrition to physiology. Metabolic and hormonal traits change during pre-partum and lactation according to parity in dairy cows, but studies of beef cows under grazing are scarce. The present study aimed to evaluate how metabolic and hormonal traits change over time, their relationship to performance, and determine if these factors differ according to the parity order of grazing beef cows. Thirty-six pregnant Nellore cows (12 nulliparous, 12 primiparous, and 12 multiparous) were used. The study started at 60 d prepartum until 203 d of lactation.

**Results:**

The initial body weight (BW) and final BW were higher for multiparous cows (*P* > 0.05). An interaction occurred between parity and day (*P* < .0001) for body condition score. Nulliparous and primiparous body condition score were reduced from − 60 prepartum to 30 postpartum, then stabilized 60 postpartum (*P* < 0.05), while multiparous maintained body condition score from − 60 days until 60 days postpartum (*P* > 0.05). Calf BW, final BW, and average daily gain did not differ between parities (*P* > 0.05). Effect of day (*P* < 0.05) was detected for non-esterified fatty acids, triglycerides, total cholesterol, LDL, VLDL, progesterone, and insulin. An interaction was observed between parity and days for glucose, HDL, β-hydroxybutyrate, creatinine and IGF-1 (*P* < 0.05). Parity affected serum urea nitrogen, total proteins, albumin, and globulins (*P* < 0.05). Parity and day relative to calving did not impact total T3 and T4 (*P* > 0.05).

**Conclusions:**

Hormonal and metabolic profile is strongly influenced by the stage of lactation. Time effects (pre-partum and lactation period) were more pronounced in nulliparous since they displayed more unbalanced metabolic and hormonal traits and lowered BCS pre- and postpartum.

## Background

Lifetime productivity of beef cows is affected by age at first calving. Beef cows are expected to begin breeding at approximately 13 to 14 months of age and calve for the first time at approximately 24 months of age as this maximizes the economic benefit of the production system [1]. However, animals are not physically or physiologically mature at this stage. So, cows experiencing their first calving are therefore in a different metabolic state than multiparous cows [2] as they require nutrients for their continued growth and the development of their calf.

In dairy cows, parity can influence the pattern of changes in metabolic hormones and metabolites following calving. However, even in dairy research in which the majority of ruminant metabolic profile studies have been conducted, published data are inconsistent [3–5].

Metabolic profile evaluation is a tool widely used in ruminant nutrition as metabolic cues that relate nutrition to physiology [6]. It helps to accurately indicate the effects of a diet [7, 8] or supplementation on animal metabolism, as well as understand homeorhetic states such as gestation and lactation [9–11] in which changes in metabolism occur to establish a new physiological state.

The metabolic processes that communicate the nutritional status of the animal are complex and result in changes in several metabolites and hormones. Non-esterified fatty acids (NEFA) and β-Hydroxybutyrate (βHB) concentrations are an index of lipid mobilization and fatty acid oxidation, so high concentrations suggest an energy deficit; while blood total protein and albumin concentrations are used as strong indicators of protein metabolism [6]. Moreover, hormones as insulin-like growth factor-I (IGF-I) and insulin are linked to both energetic and protein status [12], hence, highly associated with milk production [13] and reproduction [14]. Among the metabolic traits usually assessed, IGF-1, βHB, and NEFA concentrations are the main physiological parameters reported to be inconsistent between parity orders studies [3, 5].

While most of the scientific information regarding metabolic changes during transition period and lactation has been generated in confined systems, studies of range cattle under grazing and tropical conditions are scarce.

We hypothesized that parity influences metabolic and hormonal profile in beef cows under grazing where less mature cows display worst performance and more unbalanced metabolic traits. Therefore, the present study aimed to evaluate how metabolic and hormonal traits change over time, their relationship to performance, and determine if these factors differ according to the parity order of grazing beef cows.

## Results

The initial body weight (iBW) and final body weight (fBW) were higher for multiparous cows (*P* > 0.05). All cows average daily gain (ADG) measurements were similar between parities (*P* > 0.05). Calf iBW, fBW, and ADGs did not differ between parities (*P* > 0.05; Table 1).

**Table 1:** Performance of calves and their dams according to parity order in Nellore cows under grazing

Items	Parity				*P-*value
	Nulliparous	Primiparous	Multiparous	SEM	Par
iBW	443.0b	437.5b	515.9a	19.20	0.002
fBW	461.8b	468.3b	530.1a	17.80	0.002
ADGpre	0.41	0.46	0.34	0.059	0.373
ADGpost	−0.30	−0.18	−0.29	0.179	0.788
ADGf	0.15	0.21	0.17	0.044	0.192
Calf iBW	30.5	33.1	32.8	1.579	0.326
Calf fBW	202.9	188.9	210.0	11.78	0.306
ADGpr	0.57	0.54	0.58	0.018	0.126
ADGpo	0.76	0.75	0.77	0.039	0.821
ADGfc	0.72	0.66	0.74	0.032	0.146

*iBW* inicial body weight of the cows (kg), *fBW* final body of the cows (kg), *ADGpre* average daily gain pre-partum (kg/d), *ADGpost* average daily gain post-partum (kg/d), *ADGf* final average daily gain (kg/d), *ADGpr* average daily gain from birth to start of creep-feeding (kg/d), *ADGpo* average daily gain from start of creep-feeding until weaning (kg/d), *ADGfc* final average daily gain (kg/d)

Different letters declare significantly different between parities (*P* < 0.05)

**Fig. 1:**
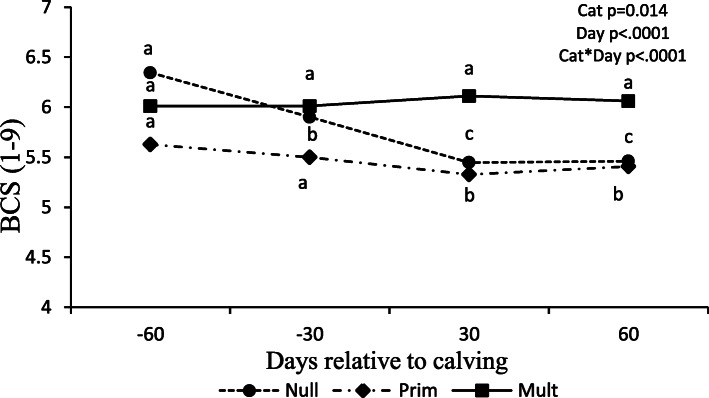
Body condition score (BCS) during of Nellore cows with different parity order under grazing according to the days relative to calving. Days followed by different superscripts within parity order are different (*P* < 0.05)

An interaction occurred between parity and days relative to calving (*P* < .0001) for body condition score (BCS). Nulliparous and primiparous BCS were reduced from − 60 prepartum to 30 postpartum, then stabilized 60 postpartum (*P* < 0.05), while multiparous maintained BCS from − 60 days until 60 days postpartum (*P* > 0.05; Fig. 1).

Milk yield (MY) was higher for multiparous (*P* < 0.05), and nulliparous and primiparous displayed similar MY (*P* > 0.05). Effect of days in milking was also detected for MY, protein and total solids (*P* < 0.05; Table 2). Milk yield of all parities was lowered at day 119 (*P* < 0.05).

**Table 2:** Milk production and composition according to parity order in Nellore cows under grazing

Items	Parity				*P-*value		
	Nulliparous	Primiparous	Multiparous	SEM	Par	Day	Par x Day
Milk yield, kg/d	6.5a	6.0a	7.2b	0.258	0.006	< 0.001	0.882
Fat^1^	5.15	4.98	5.06	0.236	0.871	0.294	0.895
Protein^1^	3.26	3.18	3.34	0.078	0.317	< 0.001	0.689
Lactose^1^	4.56	4.61	4.68	0.045	0.169	0.238	0.536
Total solids^1^	13.91	13.81	14.13	0.248	0.613	0.019	0.770

Different letters declare significantly different between parities (*P* < 0.05)

^1^g/100g

**Fig. 2:**
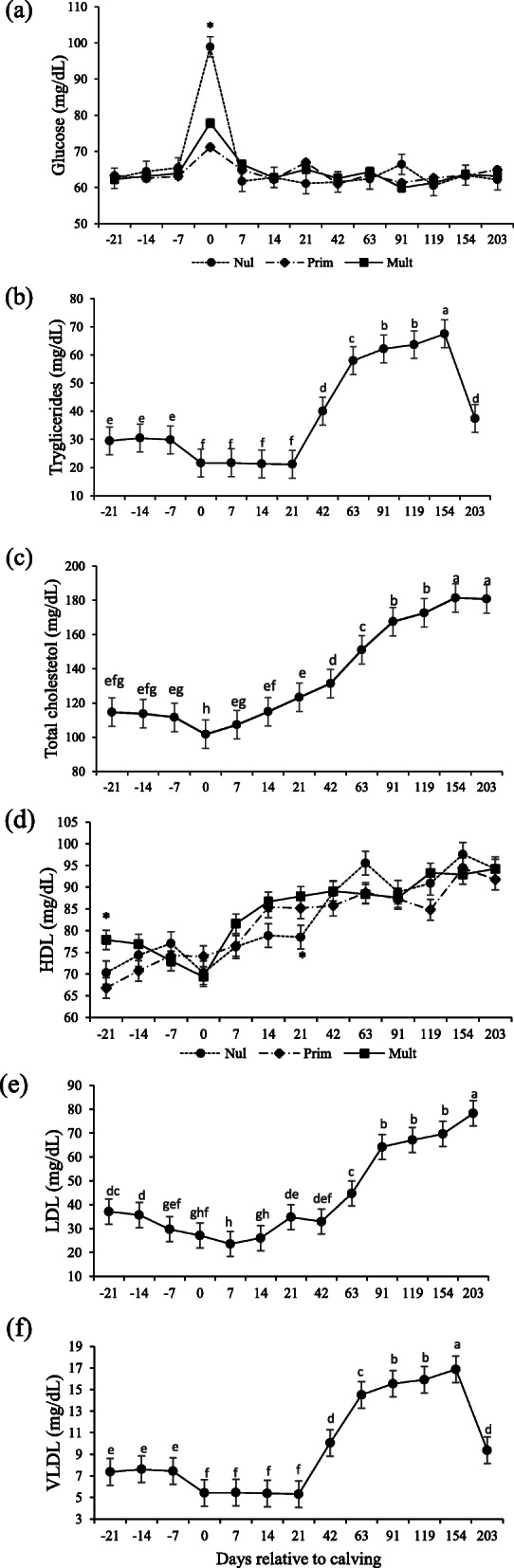
Glucose (**a**), triglycerides (**b**) total cholesterol (**c**), HDL (**d**), LDL (**e**), VLDL (**f**) serum concentrations in Nellore cows with different parity order under grazing according to the days relative to calving. Means with different superscripts differ from each other (*P* < 0.05). Days with asterisks (*) are significantly different between parities (*P* < 0.05)

An interaction occurred between parity and days relative to calving for glucose concentrations (*P* < 0.001; Table 3). Concentrations were higher for the nulliparous upon calving (*P* < 0.001; Fig. 2a).

**Table 3:** Metabolites and hormones concentrations according to parity order in Nellore cows under grazing

Items	Parity				*P-*value		
	Nulliparous	Primiparous	Multiparous	SEM	Par^2^	Day^3^	Par x Day
Glucose, mg/dL	65.66	63.93	64.32	1895	0.485	< 0.001	< 0.001
Triglycerides, mg/dL	37.47	40.35	38.60	1.450	0.332	< 0.001	0.711
Total cholesterol, mg/dL	136.49	138.14	134.31	7.842	0.868	< 0.001	0.367
VLDL, mg/dL	9.35	10.08	9.67	0.404	0.416	< 0.001	0.622
LDL, mg/dL	44.62	46.49	40.61	6.550	0.628	< 0.001	0.610
HDL, mg/dL	83.19	81.97	84.52	2.403	0.742	< 0.001	< 0.001
NEFA, mmol/L^1^	0.08	0.07	0.11	0.141	0.402	<.0001	0.936
βHB, mmol/L^1^	0.43	0.44	0.48	0.002	0.872	< 0.002	0.023
Total proteins, g/dL	6.53c	6.91b	7.24a	0.178	0.002	< 0.001	0.459
Albumin, g/dL	2.89b	2.87b	3.05a	0.067	0.034	< 0.001	0.790
Globulins, g/dL	3.77b	4.07a	4.20a	0.294	0.021	< 0.001	0.856
SUN, mg/dL	24.62a	24.78a	21.90b	1.473	0.023	< 0.001	0.206
Creatinine, g/dL	1.24	1.22	1.27	0.046	0.355	<.0001	0.028
IGF-1, ng/mL	128.73	142.08	149.18	8.194	0.184	< 0.001	0.01
Insulin, uIU/mL	2.31	2.78	2.89	0.416	0.511	< 0.001	0.816
T3, ng/mL	2.49	2.53	2.43	0.368	0.971	0.112	0.819
T4, ug/dL	8.4	7.55	7.97	0.839	0.764	0.059	0.983
Progesterone, ng/mL	5.36	4.77	4.16	1.099	0.703	< 0.001	0.537

Different letters declare significantly different between parities (*P* < 0.05)

^1^Non-esterified fatty acids (NEFA); β-hydroxybutyrate (βHB); Serum urea nitrogen (SUN)

^2^Parity (Par)

^3^Day relative to calving (Day)

Effect of day (*P* < 0.0001; Table 3), but not parity or parity and day, were detected for triglycerides, total cholesterol, LDL and VLDL (*P* > 0.05). Triglyceride decreased after calving and then remained stable up to 21 days, where it started to increase. Highest concentrations were observed at 154 days postpartum and abruptly decreased at d 203 (*P* < 0.05; Fig. 2b). Cholesterol and LDL decreased up to calving and then increased, achieving the highest values at d 203 (*P* < 0.05; Fig. 2c and d). VLDL concentrations followed the same pattern of triglyceride (Fig. 2f).

Effects of parity and days relative to calving were detected for HDL: concentrations were higher for multiparous cows at day − 21 and lower for nulliparous at day 21 (*P* < 0.0001; Fig. 2d).

**Fig. 3:**
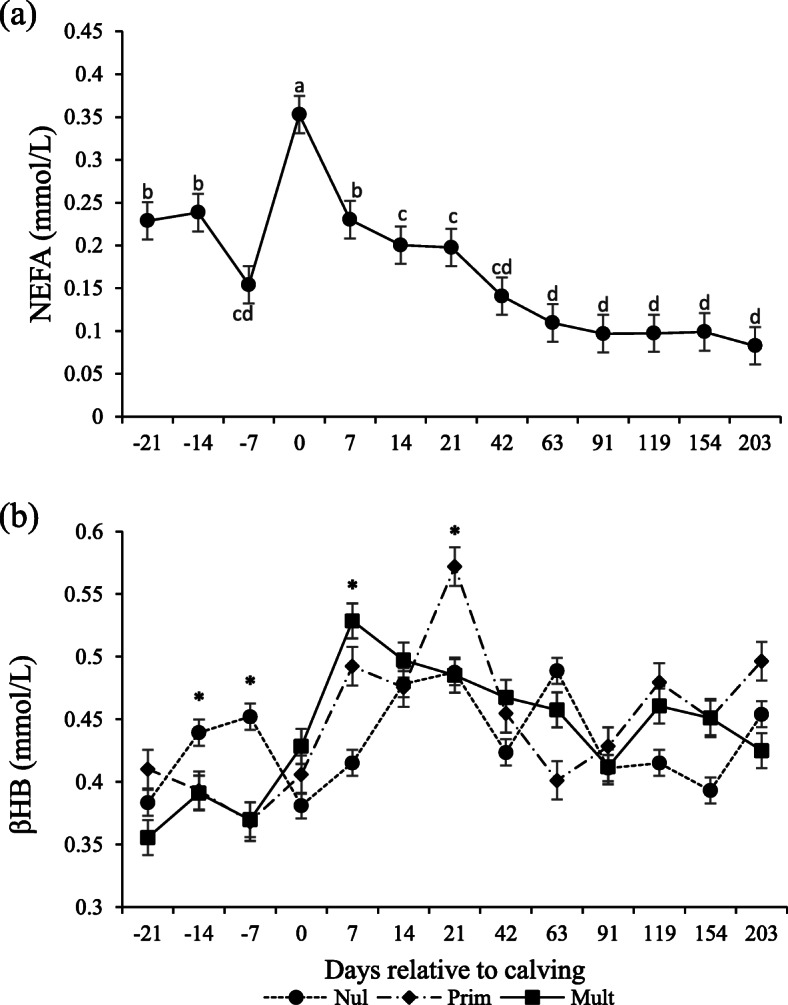
Non-esterified fatty acids (NEFA; **a**) and β-hydroxybutyrate (βHB; **b**) serum concentrations in Nellore cows with different parity order under grazing according to the days relative to calving. Means with different superscripts differ from each other (*P* < 0.05). Days with asterisks (*) are significantly different between parities (*P* < 0.05)

Effect of day (*P* < 0.0001; Table 3), but not parity or parity and day, were detected for NEFA. The NEFA serum concentrations had lower concentrations pre-partum at day − 7, then peaked at calving, lowered at 7, 14, and 21 and stabilized after day 42 (*P* < 0.05; Fig. 3a).

An interaction was observed between parity and days relative to calving for βHB (*P* < 0.020; Table 3), with higher concentrations on − 14 and − 7 days for nulliparous, higher for multiparous and primiparous than nulliparous on day 7, and higher for primiparous than the other categories at day 21 (Fig. 3b).

**Fig. 4:**
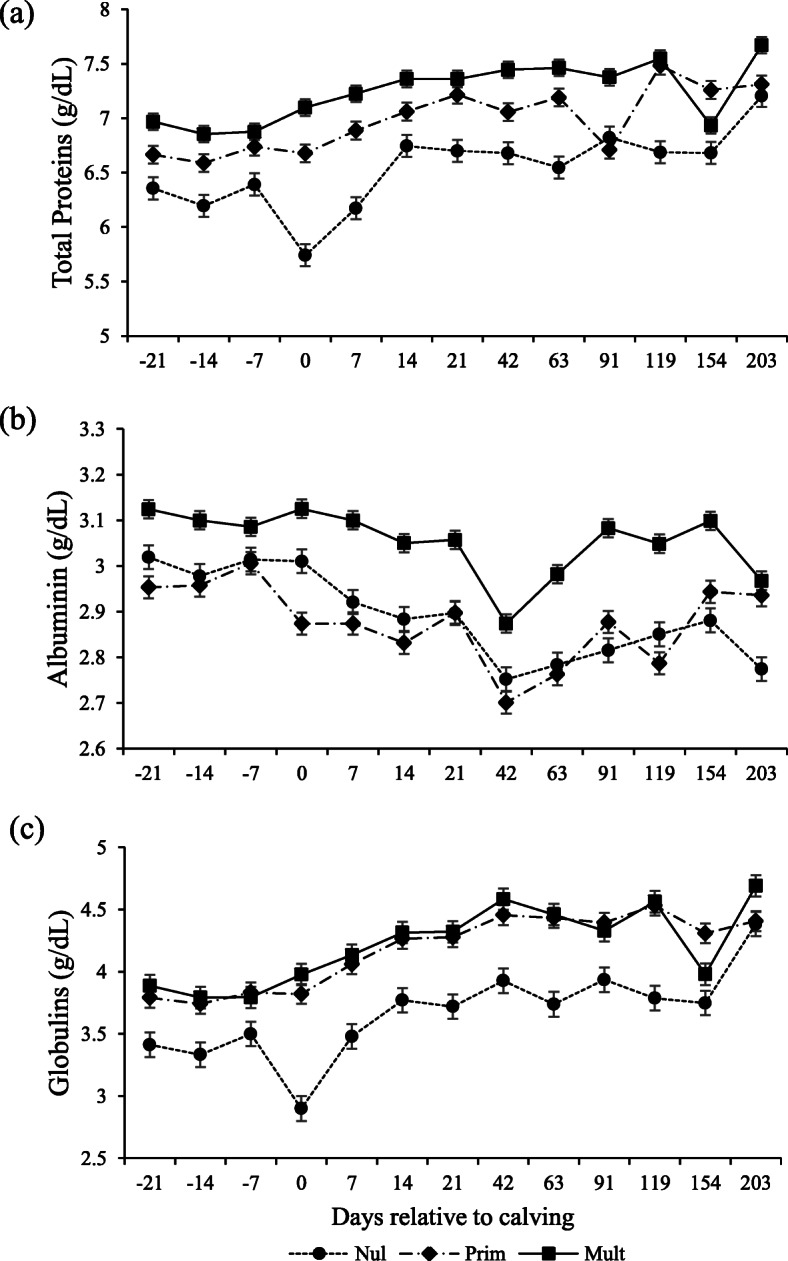
Total protein (**a**), albumin (**b**), and globulin (**c**) serum concentrations in Nellore cows with different parity order under grazing according to the days relative to calving

**Fig. 5:**
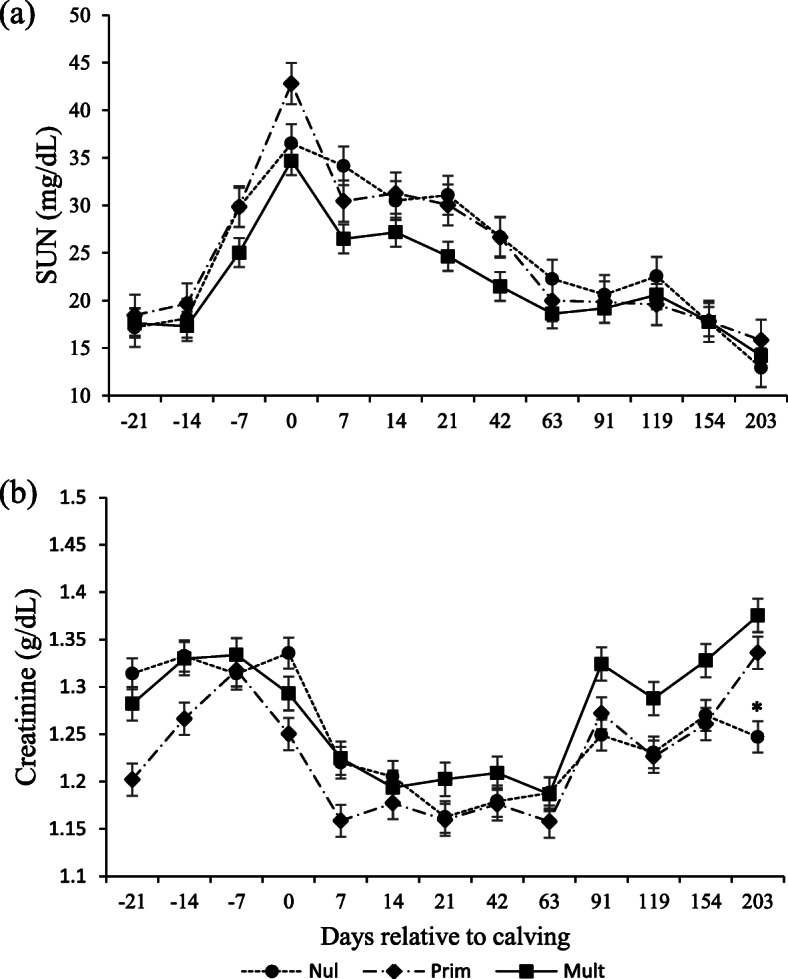
SUN (**a**) and creatinine (**b**) serum concentrations in Nellore cows with different parity order under grazing according to the days relative to calving. Days with asterisks (*) are significantly different between parities (*P* < 0.05)

Parity affected SUN, total proteins, albumin, and globulins (Table 3). Total protein increased with parity, and concentrations were highest for multiparous, followed by primiparous and nulliparous (*P* = 0.002; Fig. 4a). Globulin concentrations were higher for multiparous and primiparous than nulliparous (*P* = 0.021; Fig. 4c). Albumin was higher for multiparous than nulliparous and primiparous (Fig. 4b), whereas SUN was lower for multiparous (*P* = 0.023; Fig. 5a).

**Fig. 6:**
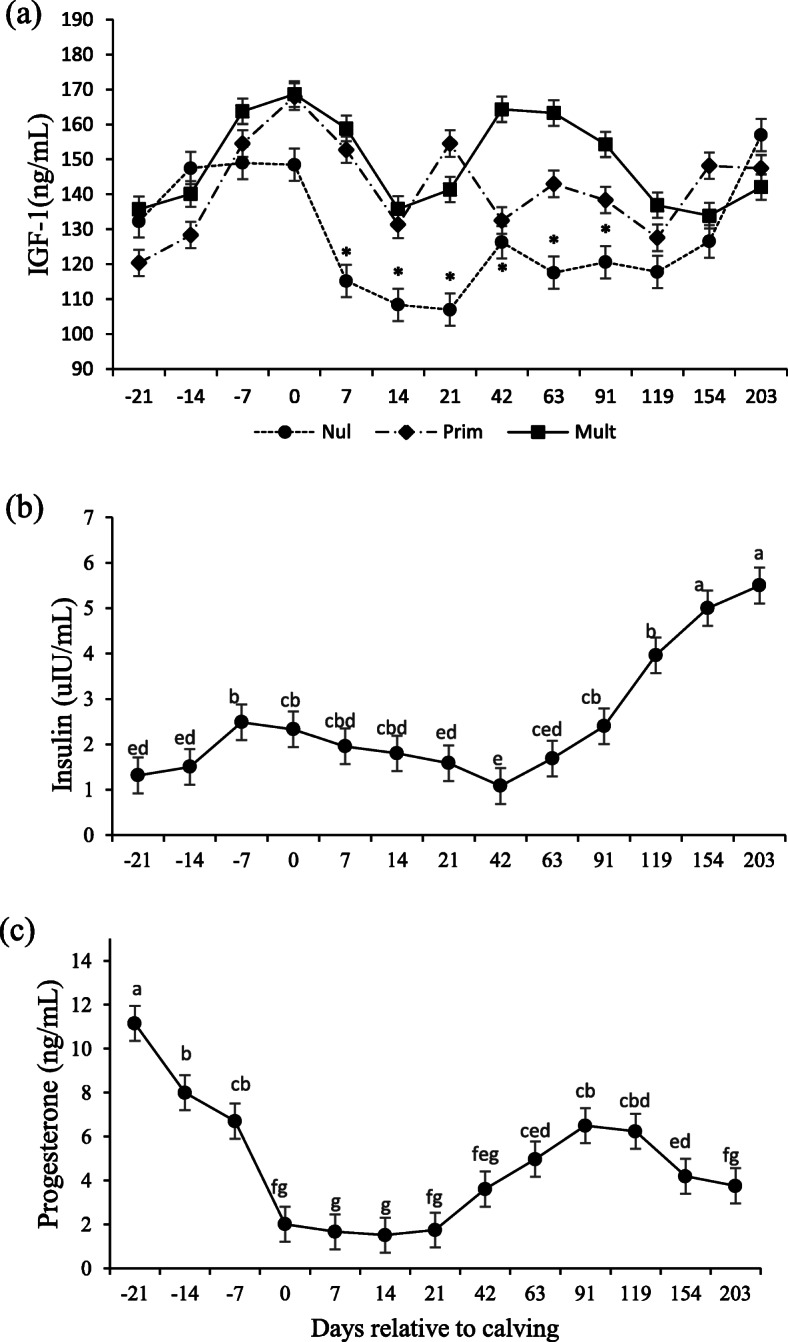
IGF-1 (**a**), insulin (**b**) and progesterone (**c**) in Nellore cows with different parity order under grazing according to the days relative to calving. Means with different superscripts differ from each other (*P* < 0.05). Days with asterisks (*) are significantly different between parities (*P* < 0.05)

An interaction occurred between parity and days relative to calving for creatinine (*P* = 0.023) and IGF-1 (*P* = 0.010; Table 3) concentrations. For creatinine, concentrations were lower for the nulliparous on day 203 (*P* < 0.05; Fig. 5b). For IGF-1, concentrations were lower for nulliparous cows at days 7, 14, and 21 and higher for multiparous than nulliparous at days 42, 63, and 91 (*P* = 0.02; Fig. 6a).

Effect of days relative to calving (*P* < 0.0001; Table 3) were detected for insulin and progesterone, where insulin concentrations peaked on day − 7, decreased until day 42 and then increased again, surpassing the first peak (P < 0.05; Fig. 6b). Progesterone levels were higher on days 91 and 119 (*P* < 0.0001; Fig. 6c).

Parity and days relative to calving did not impact total T3 and T4 concentrations (*P* > 0.05; Table 3).

## Discussion

Concentrations of glucose were similar throughout the study and maintained at a basal level, although they reached high concentrations upon calving. A high blood glucose variance is not expected since homeostatic mechanisms control blood glucose concentration [15]. However, during parturition, cortisol and epinephrine levels increase, leading to glycogenolysis [16] and thus increased blood glucose [9, 17]. In this study, nulliparous cows displayed higher glucose concentrations at calving, possibly due to more excitable temperament, stress, and fear experienced during calving than the other parities [18]. Nevertheless, cows had higher cortisol concentrations upon calving than the other days evaluated but did not show a parity effect (data not shown). Unfortunately, more physiological stress parameters are needed to infer about parity effect on stress at calving accurately, which is not our goal in this study.

NEFA concentrations peaked upon calving, and values were maintained at basal level after 42 d post-calving. Similar to glucose, heightened NEFA at parturition is likely due to the catabolic effect of stress hormones, plus dry matter intake reduction, which elicits fatty acid mobilization. It appears that hormones involved in promoting energy mobilization during stress are synergic. In adrenalectomized animals, the lipolytic response to epinephrine is reduced, indicating that glucocorticoids at least facilitate epinephrine-stimulated lipolysis. So, both cortisol and epinephrine, which are released during stress, can also influence lipid and muscle metabolism [16, 19]. Previous experiments with Nellore cows have often shown high NEFA and glucose upon calving regardless of supplementation levels [9, 17]. Besides acting as a potent lipolytic factor, epinephrine stimulates muscular glycogenolysis and amino acid output [20], which would also explain high levels of blood urea upon calving. Amino acids can be used as a gluconeogenesis source; the deamination process releases their amine groups, which will then increase and be converted to urea by the liver [15]. Moreover, muscular glycogenolysis and lipolysis may contribute to enhanced hepatic gluconeogenesis and posterior heightened blood glucose by providing lactate and glycerol, respectively, as additional substrates to the liver [15]. Parturition is indeed an intense event in which physiological changes and hormonal interactions occur that can lead to data misinterpretation in experimental conditions; so, it is worth discussing the interrelationship between metabolites and hormones during this period.

Cholesterol levels progressively increased on all postpartum days regardless of the category, a response to an increase in dry matter intake and homeorhetic changes of lactation [21]. Cholesterol levels also follow this pattern regardless of the nutritional plan in beef cows [9, 22] due to the higher need for lipoproteins to carry triglycerides to the mammary gland. Especially during this period, HDL concentrations are higher than the other lipoproteins, possibly due to either increased synthesis or VLDL catabolism by mammary tissue [23]. Therefore, the lower concentrations of triglycerides in the first weeks after calving is a combination of its utilization as energy for lactation as sources of fatty acids for milk fat synthesis [24] and depressed dry matter intake (DMI).

Increased cholesterol during postpartum could also be related to precursors being needed to synthesize steroidal hormones [21, 25]. HDL appears to be more important during this period since it participates directly in reestablishing reproductive activity. While reproductive activity is reestablished, avascularized granulosa cells are restricted to HDL cholesterol uptake [26]. Although progesterone did not differ between parities, reduced HDL concentrations of nulliparous cows at 21 days postpartum compared to the other categories could be possibly related to a delayed reestablishment of the reproductive activity. Besides HDL, differences in energy metabolism were also found for βHB.

Previous dairy cow studies have often shown higher NEFA and βHB for multiparous cows during lactation [5, 27], and they associated it with higher milk yields of this category, due to fat mobilization for milk production. However, in the current experiment, multiparous cows displayed higher βHB only during the first week postpartum, and no parity effect on NEFA concentrations. For *Bos taurus* beef cows, Sinclair et al. [10] found higher βHB postpartum for primiparous than nulliparous during the first postpartum weeks, consistent with increased βHB for primiparous at days 7 and 21. Based on the contrasting results of NEFA and βHB between studies, it seems that energy status parameters are impacted differently between parity in dairy and beef cows since they differ in milk production potential.

The BCS loss at this time explains high levels of βHB for nulliparous cows pre-calving. They suggest that this category were in a worse energy status before calving [3], which is likely to be due to higher nutritional requirements than other categories, leading to more intense energy mobilization. Nulliparous cows normally start late gestation with a good BCS since they have not been previously challenged by a gestation plus lactation. Compared to the other categories, they could not maintain BCS in the late gestation and early lactation period, even though supplementation was provided during pre-partum. Although the primary objective of this experiment was to study the metabolic and hormonal changes rather than dam reproductive efficiency, it is important to emphasize that nulliparous (primiparous after calving) have often shown lower pregnancy rates and longer postpartum intervals in livestock systems than multiparous [28–30]. Although metabolic signals mediating reproduction are not fully understood, high βHB is known to be responsible for impaired reproduction [31]. The nutritional status upon calving is the main factor influencing the length between calving and conception [32]. Elevated levels of βHB concentration pre-calving are correlated to BCS loss, and thus to delayed luteal activity [5, 33]. The levels of βHB of all parities are within the normal range for beef cows [9, 34] and do not suggest a very severe energy deficit. Rather than that, it shows that nulliparous had worst energy balance pre-calving compared to other parities, which could impact future reproduction performance.

However, ADG prepartum was similar between parities because the nulliparous is still in continuous growth, so muscle and skeletal gain might have balanced out the fat mobilization. Therefore, it was expected that the pattern of protein metabolism changes would be more intense than the energy metabolism parameters between parities.

Similarly, as presented here, studies presenting parity comparisons in dairy cows also showed increased blood total proteins with increasing parity and following the same trend with respect to the days relative to calving [35]. Total protein and albumin parameters are long-term indicators of protein metabolism [36]; thus, reduced concentrations for the less mature animals could be related to reduced protein intake. However, in this case, it is more likely due to the deviation of amino acids from albumin synthesis to other body tissues as a homeorhetic mechanism since these categories require nutrients for fetus development, lactation, and continued growth.

Multiparous cow’s albumin concentrations remained most of the time within the physiological range, but concentrations of albumin during lactation for primiparous and nulliparous were above the range (3.03–3.55 g/dL) [37]. Pronounced drop in plasma albumin above the reference values for primiparous and nulliparous may reflect a severe protein deficit [6]. On the other hand, González et al. [34] found for beef cows an overall average of plasma albumin concentration of 3.33 ± 0.407 g/dL, with extreme values between 2.18 to 3.78 g/dL.

Furthermore, it could also be assumed that the differences in blood total protein are due to different immunoglobulin concentrations between parities. The parity order impacted globulin concentrations similar to that described in several dairy cow studies: higher for multiparous and primiparous than nulliparous [35, 38]. These differences are likely due to the more mature immunological memory of older animals, (i.e., likely higher antibody titers against a broader spectrum of antigens). Globulins are not good indicators of protein metabolism and are more important as indicators of inflammatory responses and immunity. The pattern of changes in globulin concentrations during pre- and postpartum is well established in dairy cows. Authors have suggested that enhanced globulins with age are explained by a specific increase in IgG1 and IgG2, with IgG1 tending to level off and IgG2 continuing to increase, while serum IgM and IgA concentrations show no age-response [39–41]. Reduced globulin levels before calving are justified by the transfer of immunity to colostrum production [40]; so, after calving, it increases linearly, corroborating previous studies from our group [9, 17]. Despite being lower for primiparous and nulliparous, globulin values found in this study corroborates with reference limits for Holstein cows (2.5 to 6.6 g/dL) [35, 38].

To our knowledge, no studies have evaluated extensively the metabolic and hormonal traits in beef cows of different parity; therefore, most of the data herein cited for comparisons originated in studies of dairy cows.

Blood urea is considered a short-term protein indicator, and unlike the other indicators of protein status, SUN levels were lower for multiparous cows. SUN is often related to DMI and protein intake. In this case, it is very unlikely a reduced DMI for multiparous compared to the other categories since all cows were provided with the same pasture conditions and supplementation.

However, although cows had a good quantity and quality of forage available, a more intense DMI reduction postpartum may have limited energy intake for nulliparous and primiparous. If dietary energy supply is restricted, the rate of ammonia production from dietary CP exceeds the ability of the microbiota to convert it into microbial protein (lack of carbon skeleton), hence circulating ammonia concentrations will rise and be converted to urea by the liver [42]. Increased SUN can also be related to the mobilization of amino acids for gluconeogenesis, so the deamination process is responsible for the enhanced blood urea (a by-product of protein catabolism). For young cows, Sinclair et al. [10] suggested a preference for catabolism of lean tissue rather than fat tissue during the early postpartum period, although some studies did not find a difference in SUN between parities [38, 43]. Also, a likely explanation that supports these outcomes is that as multiparous cows calved with better BCS, they had more adipose tissue to support milk production, so they had to mobilize less protein to support gluconeogenesis. Therefore, these urea metabolism differences are inherent to the categories’ physiological state and the result of a possible combination of amino acid output and limited energy intake supply.

Those explanations are also supported by the lower creatinine values for nulliparous cows at the end of the study (d 203), suggesting that this category could not nutritionally overcome lactation, leading to lean tissue mobilization, which impacted muscular mass. Creatinine concentration is reported to be an index of muscle mass [44], and values slightly higher (although not significant) for nulliparous at − 21 d have been reported for dairy cows [35]. These values are explained by the higher relative muscular mass of heifers. Creatinine concentrations of all categories began to recover after 63 d postpartum. Its decreasing concentrations after parturition are due to body weight loss [9], consistent with the negative ADG postpartum until 60 d. Although changed throughout pre-partum and lactation, creatinine concentration is within the physiological range (1–2 mg/dL) [37].

These differences in energy and protein metabolism led to significantly lower milk production in first calving cows, as the differing metabolic traits may limit the partitioning of nutrients into milk. However, even receiving less milk, calves born from nulliparous cows had similar performances in comparison with other categories.

Explaining the weight gain in the calf by the cow’s milk production can be quite complex, as calves tend to consume similar quantity of metabolizable energy per unit of weight [45], which means that, if milk consumption is reduced, calf increases forage intake in attempt to meet nutritional requirements [46]. Indeed, an upcoming study (with the same animals) from our group reveals that calves born from nulliparous cows had more grazing time than calves born from older cows (Rodrigues et al., unpublished data), possibly in response to less milk ingestion, which contributed to similar ADG between the calves.

There are no conclusive results in the literature regarding the IGF-1 concentration with respect to parity. While some studies have shown higher IGF-1 for nulliparous than multiparous cows [5, 43], we found lower IGF-1 concentrations for nulliparous in the early lactation than the other categories, and values remained lower than multiparous cows until 119 days, which is consistent with Meikle et al. [3]. Even though these categories are in a different physiological state, IGF-1 concentrations during postpartum are more likely to be physiologically linked to milk production and nutritional status than growth. These results, combined with the lower BCS postpartum for nulliparous cows, reinforce that this category struggled to cope with lactation requirements. IGF-1 has an essential role in the galactopoiesis and persistency of lactation by decreasing the loss of secretory cells during lactation and by increasing cell proliferation [13, 47], which corroborates higher milk production observed in multiparous cows [4, 38]. Moreover, IGF-I is also a good indicator of the capacity to resume reproductive activity after parturition. Lower concentrations of IGF-1 for nulliparous cows during this period explains the longer postpartum interval for this category than other categories widely shown in the literature [28–30].

Many variables influencing IGF-1 concentrations can explain differences between studies, such as production system (energy intake) and genetic background. Circulating IGF-I is synthesized mainly in the liver, where its production is stimulated by the action of GH on GH receptors (GHR). Decreasing serum glucose concentrations and, consequently, insulin leads to a reduction in the GHR in the liver, the main mediator of IGF-I production [12]. Thus, the contrasting results between dairy and beef cows could also be related to the different regulation of the GH receptor in the liver. It has been shown that the liver GHR (GHR1A) is downregulated during the parturition period in dairy cows but not in beef cows [48, 49].

Both IGF-1 and insulin concentrations peaked at calving day due to increased glucose [9]. A linear increase of insulin concentrations after 42 days postpartum is related to a recovery of the DMI leading to better energy balance. Similar to insulin, both total T3 and T4 are strongly related to DMI and energy nutritional status, successfully responding to changes in beef cattle diet [7, 9, 50]. Their concentrations decrease due to energy mobilization status, slowing down the basal metabolism to lower maintenance requirements. These categories are indeed in different physiological states; therefore, the lack of differences between parities for T3 and T4 is unexpected as they are mainly responsible for basal metabolism and growth. A likely explanation is that T3 and T4 are more sensitive to changes in energy metabolism [50], while, in this study, as shown above, protein metabolism was more impacted by parity.

Thyroid hormones are galactopoietic and may play an important role in the regulation of lactation. Nevertheless, we found no significant differences in T3 and T4 concentrations during pre-partum or lactation period. Thyroid hormone concentrations throughout lactation have been found to vary in different studies. Some found no differences in T4 concentrations throughout lactation [51], while others reported that serum T4 concentrations were lower in early than in later lactation [52, 53].

Although being extremely helpful to understand changes in nutrition, there are still conflicting results about thyroid hormones regarding homeorhetic changes in metabolism of dairy and beef cows. Based on these evidences, more studies are needed to elucidate how these hormones change according to gestation and lactation in *Bos indicus* beef cows.

In summary, these underlying changes in the physiology of nutrient balance and utilization are strongly influenced by the stage of lactation. These outcomes suggest that beef cows, regardless of their parity, begin to recover their nutritional status after 42 to 63 days postpartum, based on the negative ADG until 60 d and the return of the majority of hormones and metabolites to a normal level at this time. Notably, the recovery of the nutritional status also matches the higher levels of progesterone, which is physiologically consistent, due to the reestablishment of the reproductive activity.

Late gestation and lactation homeorhetic changes affected the metabolism of the categories at different magnitudes. Although there were some differences in energy metabolism, these results suggest that the different metabolic and endocrine support between parities is more pronounced in protein metabolism. Because despite both urea and IGF-1 are also responses to the energy status, parity directly influenced all of the protein status indicators (i.e., total protein, albumin, globulins, urea, and IGF-1). Furthermore, nulliparous were more impacted by the pre-partum and lactation periods since they displayed more unbalanced metabolic and hormonal traits and lowered BCS pre- and postpartum.

## Methods

All animal care and handling procedures were approved by the Animal Care and Use Committee of the Universidade Federal de Viçosa, Brazil (protocol CEUAP-UFV 120/2018). Animals used in this study were provided by the Animal Science Department’s beef cattle farm at the Universidade Federal de Viçosa, Viçosa-MG, Brazil, where the study was conducted from July 2018 to May 2019.

### Experimental design and animals

Thirty-six pregnant Nellore cows (12 nulliparous, 12 primiparous, and 12 multiparous) were included to the study, with the following average age, BW, and BCS: 2 years, 442 ± 62 kg, 6.20 ± 0.5; 3 years, 457 ± 58 kg, 5.68 ± 0.5; 4–6 years, 505 ± 60 kg, 5.92 ± 0.5, respectively. The study started at 60 d prepartum until 203 d of lactation (2 weeks before weaning). The nomenclature for each category related to parity was set at the beginning of the experiment and used throughout the manuscript, even though after calving, the parity order changed (e.g., nulliparous became primiparous cows).

Animals were randomly divided into six paddocks, and 2 females from eachcategory were introduced into the paddocks 15 days before the beginning of the experiment to acclimate to the environment and the herd. The average area of the paddocks was 7 ha, evenly covered with *Urochloa decumbens* grass, and cows were provided free access to water and feeders.

All cows were group-fed with an energy-protein supplement (1.0 kg/d) with 35% crude protein (CP) for 60 days prepartum (Table 4). The supplement was calculated to supply approximately 40% of cow’s protein requirements, as recommended by BR-CORTE [54]. We provided a linear trough space of 0.70 m per cow to ensure homogeneous supplement intake among animals. The supplement was supplied at 1200 h.

**Table 4:** Supplement provided to cows at 60-d pre-partum and forage chemical composition

Item	^a^Supplement	*Uruchloa decumbens*			
		Dry	Dry-rainy	Rainy	Rainy-dry
DM^b^	–	384.8	270.5	266.9	258.1
OM^c^	952.8	875.8	940.4	711.7	919.2
CP^c^	36.2	63.5	81.5	90.4	78.4
NDF^c^	143.3	704.8	674.8	658	681.4
iNDF^c^	–	291.1	207.3	205.4	248.2
NDIN^d^	–	25.2	21.5	27.8	26.5

*DM* Dry matter, *OM* organic matter, *CP* crude protein, *apNDF* neutral detergent fiber corrected for ash and protein, *iNDF* indigestible neutral detergent fiber, *NDIN* insoluble neutral detergent nitrogen

^a^Supplement composition (as fed-basis): corn meal (41.2%), soybean meal (56.3%), urea:ammonium sulfate (2.5%)

^b^g/kg of natural matter

^c^g/kg DM

^d^g/kg total nitrogem

After calving, cows remained at the same paddocks, and a commercial mineral mix (CaHPO_4_ = 50.00%; NaCl = 47.775%; ZnSO_4_ = 1.4%; Cu2SO_4_ = 0.70%; CoSO_4_ = 0.05%; KIO_3_ = 0.05% and MnSO_4_ = 0.025%) was also offered to cow-calf pairs for ad libitum intake throughout the experiment, supplied separately in additional feeders. Calves were offered 5 g/kg BW of an energy-protein supplement formulated to contain 20% CP in a creep-feeding system from 90 days of age until the end of the study (d 203).

During breeding season, which started around 70 days after parturition, cows were synchronized, and fixed-time artificial insemination was performed, as a usual annual procedure of the beef cattle farm sector.

### Data collection

Cows were weighed at the beginning of the experiment (iBW; 60 d prepartum) and 7 d before the expected calving day to quantify the average daily weight gain pre-calving (ADGpre). Morevover, cows were weighed after calving, before the beginning of the breeding season (d 60) to quantify the average daily gain post-calving (ADGpost), and at the end of the experiment (fBW; d 203) to quantify the average final daily gain (ADGf). The BCS was also recorded on a scale from 1 to 9 [55], by three experienced evaluators at the beginning of the experiment (iBCS; − 60 d), − 30 d, 30 and 60 d postpartum.

Calves were weighed immediately after birth and on 2 consecutive days to determine both full and shrunk BW (14 h) at the beginning of the creep-feeding phase (d 90) and the end of the experiment (d 203). Birth weight and full BW were used to determine ADG before the creep-feeding phase (ADGpr). Shrunk BW was used to determine calves ADG from the beginning of creep-feeding to the end of the experiment (ADGpo).

### Forage sampling

Every 30 d, grass samples were collected using two methods: hand plucking to evaluate the forage selected by animals and cutting at the ground level from five delimited areas (0.5 × 0.5 m), selected randomly in each paddock to quantify total dry matter (DM) per ha. All samples were weighed, oven-dried (55 °C), then ground to pass through 1- and 2-mm screens in a Wiley mill (model 3, Arthur H. Thomas, Philadelphia, USA). All data from each month were combined and expressed as an average per season as follows: dry season = July and August (beginning of the experiment), dry-to-rainy transition season = September to November; rainy season = December to February; rainy-to-dry transition season = March to May (end of the experiment).

The average DM availability of forage was: dry season = 4.69 t/ha, dry-rainy transition = 4.33 t/ha; rainy season = 2.93 t/ha; rainy-dry transition = 3.74 t/ha. Supplement chemical composition and forage chemical composition according to the season are presented in Table 4.

### Blood sample collection

Assigning calving day as day 0, blood samples were collected from cows before feeding on days − 21, − 14, − 7, 0, 7, 14, 21, 42, 63, 91, 154, 119, and 203. Samples were collected by jugular vein puncture, using vacuum tubes with a clot activator and gel for serum separation (BD Vacutainer® SST® II Advance®, São Paulo, Brazil) to quantity serum urea nitrogen (SUN), total protein, albumin, creatinine, triglycerides, total cholesterol, high-density lipoprotein (HDL), NEFA, βHB, insulin, insulin-like growth factor (IGF-1), total triiodothyronine (T3), total thyroxine (T4), and progesterone. A tube with EDTA and sodium fluoride (BD Vacutainer® Fluorinated/EDTA, São Paulo, Brazil) was used to quantify the plasma glucose concentration. After collection, samples were centrifuged at 2200×g for 20 min. Serum and plasma were immediately frozen at − 20 °C until analyzed.

### Milk sampling

Milking was performed using a milking machine to estimate MY on days 21, 42, 63 and 119 of lactation. Milking procedures were made as described by Boggs et al. [56] which has controlled suckling period before the calf separation. To empty udders, calves were separated from their mothers from 3:00 pm to 5:45 pm, when they were reunited to dams and allowed to suckle. At 18:00 pm, calves were once again separated from dams until the next morning. At 06:00 am on the next day, cows were milked immediately after an injection of 10 UI of oxytocin (10 UI/mL; Ocitovet®, Brazil) in the mammary vein, and the produced milk was weighed. The exact time when the milking of each cow ended was recorded. Calves were kept away from their mothers until the next milking at 06:00 pm to obtain a 24-h milk production. Then, the total milk yield was calculated as the sum of both milkings. Moreover, 30 mL sample of milk from morning and afternoon milking was collected from each cow to evaluate milk composition.

### Analyses of feed, metabolites, hormones and milk

The forage and supplement samples grounded to pass through 1 mm screen were analyzed following the procedures described by the Brazilian National Institute of Science and Technology in Animal Science (INCT-CA) [57] for DM (method G-003/1), ash (method M-001/1), CP (method N-001/1), and neutral detergent fiber corrected for ash and protein (apNDF; method F-002/1). Indigestible neutral detergent fiber (iNDF) [58] was quantified in samples to pass through 2 mm using in situ incubation procedures with nonwoven textile bags (100 g/m^2^) for 288 h.

Blood concentrations of urea (K056), total protein (K031), albumin (K031), triglycerides (K117), total cholesterol (K083), HDL (K071), and glucose (K082) were determined using Bioclin® kits (Belo Horizonte, Brazil). NEFA and βHB were analyzed using Randox® kits (FA115 and RB1007, Antrim, UK). All the above-mentioned analyses were determined by chemiluminescence method in an automated biochemical analyzer (Mindray, BS200E, Shenzhen, China). Insulin, total T3, total T4, and progesterone were analyzed by Beckman Coulter® kits (33,410, 33,830, 33,800, and 33,550, Brea, USA). IGF-1 contents were quantified using Siemens® kits (Berlin, Germany) in an automated chemiluminescence analyzer (Berlin, Germany). The intra- and inter-assay CV were, respectively, 2.3 and 4.5% for insulin, 4.1 and 5.9% for T3, 3.7 and 4.8% for T4, 3.8 and 5.6% for IGF-I, and 6.8 and 8.1% for progesterone.

The serum contents of low-density lipoprotein (LDL) and very-low density lipoprotein (VLDL) were calculated by the equation: TC = HDL + LDL + VLDL, where TC = total cholesterol and VLDL = triglycerides/5 [59]. Globulins were calculated by the difference between total proteins and albumin. SUN was estimated as 46.67% of total serum urea.

Milk samples were analyzed regarding protein, fat, lactose, and total solids contents using infrared spectroscopy (Foss MilkoScan FT120, São Paulo, Brazil).

### Statistical analyses

The basic statistical model was used as follow:

$${Y}_{ijk}=\mu +{P}_i+{C}_j+{e}_{(ij)k}$$

where: Y_ijk_ = observation taken on animal k, pertaining to parity j, within paddock i; μ = overall constant; P_i_ = paddock effect I (random); C_j_ = Category (parity) effect j (fixed) e_(ij)k_ = random effect, unobservable, assumed to be NIID (0, σ^2^_e_);

Blood parameters, milk yield and BCS taken over time in the same animals were evaluated as repeated measurements, where the best structure of (co) variance matrix was chosen based on Akaike’s information criterion with correction. Effects of parity, day, and parity and day interaction were analyzed. When necessary, means were compared by Fisher’s least significant difference. The degrees of freedom were estimated using the Kenward-Roger method. The analyzes were performed using the PROC GLIMMIX of the Statistical Analysis System (SAS). All the statistical evaluations were performed considering 0.05 as the critical level of probability for the occurrence of the type I error.

## Data Availability

The data generated during the current study are available from the corresponding author on reasonable request.

## Abbreviations

ADGpre: Average daily gain pre-partum
ADGpost: Average daily gain postpartum
apNDF: Neutral detergent fiber corrected for ash and protein residue
BCS: Body condition score
βHB: Beta-hydroxybutyrate
SUN: Serum urea N
BW: Body weight
CP: Crude protein
DM: Dry matter
HDL: High density lipoprotein
LDL: Low density lipoprotein
IGF-1: Insulin-like growth factor
iNDF: Indigestible neutral detergent fiber
MM: Mineral mixture
MY: Milk yield
NDF: Neutral detergent fiber
NEFA: Non-esterified fatty acid
VLDL: Very-low density lipoprotein
OM: Organic matter
T3: Total triiodothyronine
T4: Total thyroxine
